# HHV-6 Infection in an Immunocompetent Patient with Multi-organ Failure

**DOI:** 10.7759/cureus.5475

**Published:** 2019-08-24

**Authors:** Richard N Wang, Radhames Ramos De Oleo, Sushilkumar S Gupta, Domingo Y Ynoa Garcia, Yizhak Kupfer

**Affiliations:** 1 Emergency Medicine, Maimonides Medical Center, Brooklyn, USA; 2 Critical Care, Maimonides Medical Center, Brooklyn, USA; 3 Cardiology, Cardiology Unlimited, New York, USA

**Keywords:** hhv-6, encephalitis, immunocompetent

## Abstract

Human herpesvirus 6 (HHV-6) is a double-stranded DNA virus part of the Herpesviridae family that colonizes nearly 100% of the human population. The virus is known to be the etiologic agent of roseola infantum, a self-limited disease in childhood and reactivation of the virus later in life is linked to potentially severe manifestations, including encephalitis, in immunosuppressed patients. It is rare in immunocompetent patients, but there have been several reports of encephalitis due to HHV-6 reactivation. We describe here a case of altered mental status and multi-organ failure in an immunocompetent woman, whose cerebrospinal fluid (CSF) was positive for HHV-6 DNA by polymerase chain reaction (PCR).

## Introduction

Human herpesvirus 6 (HHV-6) is a double-stranded DNA virus part of the Herpesviridae family. All herpesviruses can establish a lifelong latent infection after the viral genome integrates into host cell DNA. HHV-6 colonizes nearly 100% of the human population [1]. The virus is known to be the etiologic agent of roseola infantum, a self-limited disease in childhood. Reactivation of the virus later in life is linked to potentially severe manifestations, including encephalitis, in immunosuppressed patients, such as after hematopoietic stem cell transplant [2]. It's occurance is rare in immunocompetent patients, but there have been several reports of encephalitis due to HHV-6 reactivation [3]. We report the case of altered mental status and multi-organ failure in an immunocompetent woman, whose cerebrospinal fluid (CSF) was positive for HHV-6 DNA by polymerase chain reaction (PCR).

## Case presentation

A 93-year-old woman with history of hypertension, diabetes mellitus, coronary artery disease, and atrial fibrillation presented with fever, altered mental status, and reported seizure-like activity. She was, at baseline, able to ambulate with a walker. She had been seen the previous day at an outside hospital for a mechanical fall, with negative computed tomography (CT) scan of the head. The patient was discharged early in the morning and later that morning was unable to be woken up, with the family noting seizure-like activity. She was subsequently brought to the emergency department.

The patient was febrile (temperature 100.4), tachycardic (heart rate between 90-110s), hypoxic (peripheral oxygen saturation 88% on 2 liter nasal cannula), and noted to have a left-sided paresis on physical exam. Her initial National Institute of Health (NIH) Stroke Scale score was 24. Laboratory results were significant for creatinine (1.7 mg/dl; unknown baseline), troponin (3.21 ng/ml), and lactic acid (4.7 mmol/L). Non-contrast CT of the head was negative. CT scan showed bilateral lobar pneumonia, dilated esophagus with a debris level (but not distal stricture that would point towards achalasia), and asymmetric opacification of the middle cerebral artery branches without corresponding asymmetry on CT perfusion. She was deemed not a candidate for tissue plasminogen activator or endovascular revascularization.

The patient was intubated for airway protection and admitted to the medical intensive care unit for altered mental status, cerebrovascular accident (CVA), non-ST elevation myocardial infarction (NSTEMI), and respiratory failure secondary to bilateral aspiration pneumonia. She was started on aspirin, high intensity atorvastatin and minocycline for CVA, levetiracetam for seizure prophylaxis, and intravenous vancomycin, ceftriaxone, metronidazole and ampicillin for broad coverage given possible meningitis as well as aspiration pneumonia. An EEG was performed with no evidence of seizure activity. A lumbar puncture was performed by interventional radiology on day five of admission and revealed in the CSF 14 white blood cells/μl (86% lymphocytes, 14% neutrophils), 155 red blood cells/μl, glucose 82 mg/dl, and protein 50 mg/dl; no opening pressure was documented. HHV-6 DNA was detected in the CSF, viral load was 395 copies per ml. A Magnetic Resonance Imaging (MRI) was never performed. Given the patient’s gradually improving mental status, antiviral treatment was deferred. The patient was extubated on day ten of admission and transferred to the medical floor, where she was ultimately discharged to hospice care per the family’s wishes.

## Discussion

HHV-6 is well known to cause roseola infantum and clinical manifestations in immunosuppressed patients, in particular after hematopoietic stem cell transplant. However, HHV-6 meningoencephalitis in immunocompetent patients is rare, although there are increasing numbers of reported cases. The presumed pathogenesis is reactivation of the virus. It has been established that HHV-6 has a tropism for central nervous system tissues [4]. One study retrospectively analyzed samples from patients with HHV-6 by PCR from various tissues, including blood, CSF, ascites, and tissue biopsy [5]. Three types of clinical entities were identified: neurological manifestations, including convulsions and encephalitis in non-immunocompromised patients, digestive problems in immunosuppressed patients, and severe maternal-fetal infection. The encephalitis may potentially present as status epilepticus [6].

The significance of HHV-6 in the CSF has been debated. It is difficult to distinguish between latent and active viral infection. The high CSF viral load supports the possibility of HHV-6 as the etiologic agent of disease in the case series by Isaacson et al [7]. There is increased levels of HHV-6 IgG and IgM in a subset of encephalitis patients compared with other neurological diseases [8]. On the other hand, HHV-6 has been detected in brain tissue specimens of healthy patients with no evidence of neurological disease [9]. In addition, reports of HHV-6 encephalitis have had differing findings on neuroimaging, illness severity and outcome, and hospital course [3]. However, this may be explained by viral genome variations and host genetics. In our case, we might debate that the patient had other causes for the alteration in her mental status but despite this, we cannot ignore the high viral load of HHV-6 in the CSF. The other conditions that the patient had that could have altered her mental were not as definitive as to explain her profound delirium, only the active infection of a neurotropic virus could explain such findings

There are currently no therapies approved for treatment. Small studies and case reports describe success with drugs such as cidofovir, ganciclovir, foscarnet, and new small molecule inhibitors are being developed [10]. For example, there was a significant decrease in viral load after treatment with ganciclovir. However, there is a lack of definitive cause and effect relationship between HHV-6 reactivation and disease that requires intervention. In addition, the efficacy of ganciclovir and other compounds may not be optimal and is linked to toxicity. Valganciclovir has been successful and suggested as a potential practical outpatient treatment [10]. In our patient or any other patient, even if other conditions that can explain acute changes in mental status, it is important to maintain neurological infections with HHV-6 in our list of differential diagnoses

## Conclusions

We believe this case report will help physicians to consider HHV infection early in the list of differentials of altered mental status and in the management of this patient. Even if the HHV-6 virus is a co-condition and other conditions in the patient could explain alteration in sensorium, we should keep this virus as a differential.
